# Folic acid supplementation during pregnancy and associations with offspring size at birth and adiposity: a cohort study

**DOI:** 10.1186/s13104-021-05575-y

**Published:** 2021-04-30

**Authors:** Clive J. Petry, Ken K. Ong, Ieuan A. Hughes, David B. Dunger

**Affiliations:** 1 Department of Paediatrics, University of Cambridge, Cambridge Biomedical Campus, Hills Road, Cambridge, CB2 0QQ UK; 2 MRC Department of Epidemiology, University of Cambridge, Cambridge, UK; 3 Institute of Metabolic Science, University of Cambridge, Cambridge, UK

**Keywords:** Pregnancy, Growth, Development, Gestational diabetes

## Abstract

**Objective:**

Previously we observed that maternal multiple micronutrient supplementation in pregnancy was associated with increased offspring size at birth and adiposity, as well as with maternal gestational diabetes risk, in the Cambridge Baby Growth Study. In this study we therefore investigated whether folic acid supplementation specifically is associated with similar changes, to test the hypothesis that folic acid supplementation mediates such changes.

**Results:**

The majority of mothers who reported supplementing with folic acid in pregnancy (n = 776 in total, 526 of which took multiple micronutrient preparations) did so either from pre- (n = 139) or post-conception (n = 637) largely for all or just the first half of pregnancy. A minority of mothers (n = 198) reported not supplementing with folic acid. Folic acid supplementation in pregnancy was not associated with birth weight [β’ = − 0.003, p = 0.9], height [β’ = − 0.013, p = 0.6], head circumference [β’ = 0.003, p = 0.09] or adiposity (ponderal index [β’ = 0.020, p = 0.5], skinfolds thicknesses [β’ = − 0.029 to + 0.008, p = 0.4–0.9]). Neither was it associated with the development of maternal gestational diabetes (risk ratio 1.2 [0.6‒2.2], p = 0.6). These results suggest that folic acid supplementation in pregnancy did not mediate the previously observed increases in offspring size at birth and adiposity, or the raised gestational diabetes risk, in response to supplementation with multiple micronutrients.

## Introduction

Multiple micronutrient supplementation in pregnancy leads to a reduced risk of the baby being born with a low birth weight, and possibly one for being born small for its gestational age (SGA) [1]. Consistent with this, we recently studied the effects of multiple micronutrient supplementation in pregnancy in the prospective, longitudinal Cambridge Baby Growth Study (CBGS) and found that it was associated with increased size (including weight and head circumference) and adiposity (skinfold thicknesses in the flank, subscapular and triceps regions) at birth [2]. In the mothers it was associated with increased risk of developing gestational diabetes (GDM), and most of the associations with increased offspring size at birth and adiposity were somewhat attenuated by adjusting the statistical models for GDM. This suggests a role for the processes involved in increasing offspring size at birth in GDM pregnancies [3] in possibly mediating the increased size at birth and adiposity in offspring whose mothers supplemented with multiple micronutrients in pregnancy.

In iron replete women in the U.K. the only micronutrients that are specifically recommended to supplement dietary intake with during pregnancy (and whilst attempting to get pregnant) are folic acid (at a dose of 400 µg per day) and, in certain months of the year, vitamin D [4]. Folic acid supplementation in pregnancy, often taken as part of multiple micronutrient preparations, has been associated with both increased [5, 6] and decreased [7] risk of GDM in the mother, and decreased risk of SGA in the offspring [8] in previous studies. We therefore investigated the effects of folic acid supplementation in pregnancy on offspring size at birth and adiposity, as well as adverse pregnancy outcomes on the mother in the CBGS, testing the hypothesis that our previous findings with multiple micronutrient supplementation [2] were mediated specifically by folic acid supplementation.

## Main text

### Methods

#### Cambridge baby growth study

The initial phase of the CBGS involved recruiting 2229 participants from 2001–9 and has been described in detail elsewhere [9]. At recruitment from the booking clinic at the Rosie Maternity Hospital, Cambridge, U.K., each participant was given an extensive questionnaire to fill in as pregnancy progressed [10]. Around week 28 of pregnancy 1074 of the women underwent standard 75 g oral glucose tolerance tests after fasting overnight, and GDM status was defined from these, as previously described [11]. Blood pressure measurements across pregnancy (plus diagnoses related to high blood pressure) were collected retrospectively from hospital notes in 720 of the participants to increase the amount of useful available information; gestational hypertension status was defined from these as previously described [12].

From all the women recruited to the CBGS during this time the following were excluded from the present analysis: those that had already withdrawn from the study before the birth of their baby, twin pregnancies (because of the impact of multifetal pregnancies on offspring size at birth), and those that did not fill in (specifically the questions about maternal supplement intake during pregnancy) and return their pregnancy questionnaire.

Offspring birth weights were recorded from hospital notes. Other newborn measurements (length, head circumference, skinfold thickness at flank, quadriceps, subscapular and triceps regions) were made by trained paediatric nurses as soon as possible after birth [at a median (inter-quartile range) age of 2 (1–16) days], as described [13]. Low birth weight, SGA, and pre-term status were also defined as described [2]. The body mass index (BMI) was calculated as the body weight (pre-pregnancy for the mother) divided by the height or length squared, and the ponderal index calculated as the body weight divided by the length cubed. Pregnancy weight gain was calculated as the maternal pre-pregnancy weight subtracted from the pregnancy weight (from the final week of pregnancy), both of which were collected from the pregnancy questionnaire.

This analysis, using results from the CBGS cohort, was run in an unmatched exposed v. non-exposed format as per that of our multiple micronutrient supplementation study [2]. The exposed group contained women who either supplemented with multiple micronutrients in pregnancy that included folic acid or supplemented with folic acid specifically (largely either in isolation or in combination with iron later in pregnancy). Folic acid supplementation dosage (both daily and total) in this group was calculated using the questionnaire (checking the folic acid content of the reported supplement used on the internet), plus the length of reported time that the folic acid was supplemented. The non-exposed group contained women who either did not take dietary supplements in pregnancy, or who took supplements that did not include folic acid (e.g. iron in isolation, vitamin C, or vitamin D with or without added calcium).

#### Statistical analysis

Risk ratios of adverse pregnancy outcomes by folic acid supplementation were analysed by log-binomial regression. Other categorical variables were analysed using χ^2^ or Fisher’s exact tests (as appropriate). Continuous variables were analysed using linear regression, adjusted for confounders where appropriate. Where the dependent variable residuals were skewed, the models were analysed with prior transformation of the data so that they were normally distributed. Missing data were treated by case or listwise deletion. P < 0.05 was considered statistically significant throughout. Stata (version 13.1; Stata Corp., from Timberlake Consultants Ltd., Richmond, Surrey, U.K.) was used to perform the statistical analyses.

### Results and interpretation

**Fig. 1:**
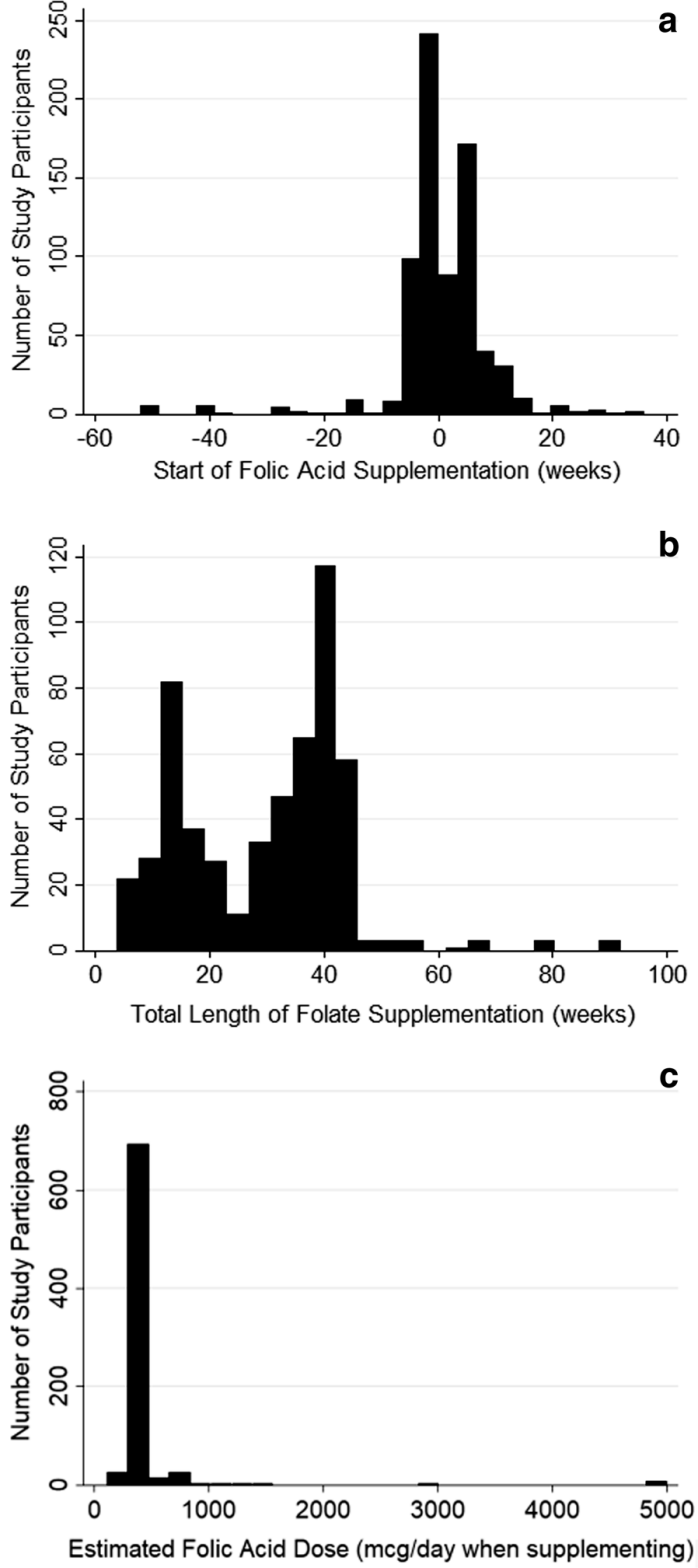
Participant numbers by the **a** start, **b** length and **c** dose of folic acid supplementation

This analysis included 974 pregnancies: 776 with mothers that supplemented with folic acid and 198 that did not. Of those that supplemented with folic acid, most (n = 526) did so via the form of multiple micronutrient supplementation (Table 1). In terms of when they started supplementing, there was a bimodal distribution (Fig. 1a). This may have reflected one group of women who presumably were planning a pregnancy and therefore started supplementing prior to its onset, as recommended [4], and another group of women who started to supplement as soon as they knew that they were pregnant or as soon as the concept of supplementing came to their attention. The total length of time that women supplemented was also bimodal (Fig. 1b): one group of women seemed to supplement with folic acid throughout pregnancy, while another group tended to supplement for up to half of pregnancy (as recommended [4]). The modal dose of folic acid supplemented with was 400 µg/day (again as recommended [4]) (Fig. 1c).

**Table 1:** A comparison of the characteristics of CBGS participants by folic acid supplementation in pregnancy

Maternal Characteristic	No folic acid supplementation	Folic acid supplementation	p-value
Supplemented with multiple micronutrients (all of which contained folic acid)	0 yes 198 no	526 yes 250 no	< 0.001
Age (years)	33.1 (32.5–33.7) (n = 179)	33.6 (33.3–33.9) (n = 710)	0.2
Height (m)	1.65 (1.64–1.66) (n = 180)	1.66 (1.66–1.67) (n = 729)	0.2
Pre-pregnancy weight (kg)	67.0 (65.0–69.0) (n = 177)	66.1 (65.1–67.1) (n = 708)	0.4
Weight gain in pregnancy (kg)	7.5 (6.3–8.6) (n = 121)	8.7 (8.2–9.3) (n = 523)	0.1
Pre-pregnancy BMI (kg/m^2^)	24.5 (23.9–25.2) (n = 172)	24.0 (23.6–24.3) (n = 693)	0.1
Smoked during pregnancy	11 yes 184 no	20 yes 742 no	0.03
Parity (n of pregnancies)	2.0 (1.9–2.1) (n = 195)	1.7 (1.6–1.8) (n = 761)	< 0.001
Anaemia	3 yes 183 no	19 yes 717 no	0.6
GDM	11 yes 112 no	57 yes 489 no	0.6
Pre-eclampsia	3 yes 195 no	12 yes 764 no	1.0
Gestational hypertension	7 yes 78 no	21 yes 366 no	0.3
Premature birth (< 37 weeks gestation)	3 yes 192 no	14 yes 748 no	1.0
Length of pregnancy (weeks)	39.9 (39.7–40.1) (n = 195)	39.9 (39.8–40.0) (n = 762)	1.0
Offspring low birth weight	5 yes 190 no	21 yes 739 no	0.9
Offspring SGA at birth (n no/yes)	0 yes 195 no	5 yes 755 no	0.6

Data are mean (95% confidence interval) or number of participants

Most of the maternal clinical characteristics did not differ between those women that supplemented and those that did not (Table 1). However, the average parity was lower in those that supplemented with folic acid; also, proportionally fewer smoked in pregnancy in those that supplemented (in a cohort where smoking in pregnancy was rare [9]). Folic acid supplementation in pregnancy was not associated with any of the adverse outcomes of pregnancy that were tested [mean risk ratio (95% confidence interval)]: GDM 1.2 (0.6‒2.2), gestational hypertension 0.7 (0.3‒1.5), pre-eclampsia 1.0 (0.3‒3.6), anaemia 1.6 (0.5‒5.4), pre-term birth 1.6 (0.5‒5.4), low birth weight 1.1 (0.4‒2.8).

The lack of association with GDM risk is inconsistent with other studies investigating links between folic acid supplementation and risk of GDM but results from these studies are themselves inconsistent with both higher [5, 6] and lower [7] risk being reported. One study found that the risk for GDM was both dose- and supplementation-length dependent [14]. Results from the current study are inconsistent with this, however, as the dose of folic acid supplemented with (either expressed as the daily dose or the total dose) or duration of supplementation were also not associated with GDM [all risk ratios (95% confidence intervals): 1.0 (1.0‒1.0)]. This inconsistency may be due to the lack of participants in the present analysis who supplemented with high dose folic acid [14]. Results from previous studies seeking associations between folic acid supplementation in pregnancy and blood pressure-related outcomes have been equivocal. Consistent with the results that we observed, one meta-analysis shown no associations between folic acid supplementation in pregnancy and gestational hypertension or pre-eclampsia [15]. Another meta-analysis, however, found such supplementation to be associated with a reduced risk of pre-eclampsia [16]. Previous results are also equivocal for associations between folic acid supplementation in pregnancy and risk of preterm birth, with two meta-analyses showing no association [17, 18], like we observed, and others showing a reduced risk of preterm birth [19, 20]. One meta-analysis also found folic acid supplementation in pregnancy to be associated with a reduced risk of low birth weight, although only at high doses [21]. Given that in our analysis the bulk of the women who supplemented with folic acid did so in the form of multiple micronutrient supplements, where the dose of folic acid was generally that recommended for pregnant women rather than higher doses, our results are therefore not inconsistent with this.

Folic acid supplementation in pregnancy was not associated with any index of offspring size at birth or adiposity (Table 2). Once again data on this subject published in the literature have been conflicting. Two meta-analyses found no association with birth weight [17, 21] but an increase in birth weight was found in two other meta-analyses [18, 22], one of which was in a dose-dependent manner [18]. No previous studies in humans have investigated offspring adiposity at birth where mothers supplemented their diets with folic acid in pregnancy. In the present analysis, given that maternal folic acid supplementation failed to show associations with any of the other markers of size at birth, it is not surprising that there were no significant associations with either BMI (or ponderal index) or any of the skinfold thicknesses.

**Table 2:** Associations between folic acid supplementation status in pregnancy and indices of offspring size at birth

Measure	No maternal Folic acid supplementation	Maternal Folic acid supplementation	Standardised β (×100)	p-value
Weight (kg)	3.5 (3.4–3.6) (n = 172)	3.5 (3.5–3.5) (n = 689)	− 0.3 (− 5.9 to 5.2)	0.9
Length (cm)^a^	51.5 (51.2–51.8) (n = 165)	51.4 (51.3–51.6) (n = 674)	− 1.3 (− 6.3 to 3.6)	0.6
Head Circumference (cm)^a^	35.3 (35.1–35.5) (n = 166)	35.3 (35.2–35.4) (n = 674)	0.6 (− 4.3 to 5.5)	0.8
BMI (kg/m^2^)^a^	13.1 (12.9–13.3) (n = 165)	13.2 (13.1–13.3) (n = 672)	1.0 (− 4.4 to 6.3)	0.7
Ponderal Index (kg/m^3^)^a^	25.5 (25.1–25.9) (n = 165)	25.7 (25.5–25.8) (n = 672)	2.0 (− 3.2 to 7.1)	0.5
Flank skinfold thickness (mm)^a^	6.2 (6.0–6.5) (n = 166)	6.1 (6.0–6.2) (n = 673)	− 2.9 (− 8.8 to 3.5)	0.4
Quadriceps skinfold thickness (mm)^a^	8.0 (7.6–8.3) (n = 166)	8.0 (7.8–8.1) (n = 673)	− 0.4 (− 5.8 to 5.0)	0.9
Subscapular skinfold thickness (mm)^a^	5.4 (5.2–5.6) (n = 166)	5.4 (5.3–5.5) (n = 673)	− 2.0 (− 8.1 to 4.1)	0.5
Triceps skinfold thickness (mm)^a^	5.5 (5.3–5.7) (n = 166)	5.5 (5.4–5.6) (n = 673)	0.8 (− 5.4 to 7.1)	0.8

Data and standardised βs are presented as mean (95% confidence interval)

All models adjusted for gestational age at birth, parity, smoking during pregnancy, offspring sex and maternal pre-pregnancy BMI

^a^Models additionally adjusted for age at assessment

In conclusion, in the present analysis we could not find any associations between folic acid supplementation in pregnancy and either adverse outcomes of pregnancy or markers of offspring size at birth or adiposity. This is not surprising given that the literature contains highly conflicting studies in this area. Although peri-conceptional folic acid supplementation remains the most suitable preventative measure to lower risk of neural tube defects in the offspring [23], in this analysis we could find no evidence to suggest that the increases in offspring size at birth and adiposity in women that supplemented with multiple micronutrients in pregnancy might have resulted specifically from folic acid supplementation.

## Limitations

- Folic acid supplementation in pregnancy was self-reported, which could have led to inaccurate categorisation.
- Dietary folic acid intake was not recorded, so the folic acid supplementation doses may not have borne much resemblance to the total daily folic acid intakes.
- As the exposed group in this analysis contained both women who supplemented their diets with folic acid in isolation and those whose folic acid was included in multiple micronutrient preparations, the negative results could have been affected by confounding effects of other micronutrients. If folic acid had mediated the previously observed significant associations with multiple micronutrient supplementation [2], however, it would have been expected that the rationale used in this analysis would have strengthened these associations not attenuated them.
- Missing data were manifest for most of the variables and were dealt with by case or listwise deletion. In particular, missing data related to the supplementation with folic acid during pregnancy (e.g. missing due to the lack of filling in the pregnancy questionnaire in the section related to supplementation, or not returning it) could have introduced biases to the study.
- The lack of significant statistical associations observed could theoretically be caused by insufficient statistical power if the true effect sizes were very small (i.e. a type II statistical error). However, they still clearly do not explain the significant associations detectable with multiple micronutrient supplementation in pregnancy [2].

## Data Availability

The dataset analysed during the current study is available in the University of Cambridge Apollo repository, https://doi.org/10.17863/CAM.59641.

## Abbreviations

BMI: Body mass index
CBGS: Cambridge Baby Growth Study
GDM: Gestational diabetes
SGA: Small for gestational age
